# Memoir on the Simple Ulcer of the Stomach

**Published:** 1856-06

**Authors:** 

**Affiliations:** Professor of Pathological Anatomy of the Medical Faculty of Paris


### *Memoir on the simple Ulcer of the Stomach.*

A Paper read before the Institute of France by Mr. Cruveilhier, Professor of Pathological Anatomy of the Medical Faculty of Paris.

The subject of the memoir which I now lay before the Academy is a peculiar disease of the stomach, which is often mistaken in practice for the cancerous affection of the same organ, sometimes for gastralgia, or the various forms of chronic gastritis.

This disease, or rather this lesion, the precise nature of which could be ascertained by pathological anatomy, is styled in the following pages the simple ulcer, or chronic simple ulcer of the stomach, in order to indicate on the one hand, its form which is that of an ulcer, generally chronic, and on the other hand its benignity, in opposition to the incurability and malignancy of the cancerous ulcer of the stomach. A general description of the simple ulcer of the stomach must comprehend,

1. Its anatomical character, in order that it may be classed among the different varieties of disease.
2. Its clinical character, which must teach us how to recognize this disease at the bed side of the patient.
3. Its therapeutical character, which will establish not only its curability, but even the tendency which nature has to perform a cure, provided all irritating treatment be avoided.

*Anatomical character.*—Anatomically considered, the simple ulcer of the stomach presents a loss of substance, generally circular with an abrupt, indurated edge, varying in size from one to several lines in diameter.

The simple ulcer is generally solitary, occupying for the most part either the posterior parietes of the stomach, or its lesser curvature, and as a general thing it is closer to the pyloric than to the cardiac orifice; sometimes it occupies the pylorus itself, presenting a sort of circular zone.

The simple ulcer of the stomach extends itself at the same time in depth and surface, and when it has overcome the resistance opposed by the fibrous tunic of the stomach, then the muscular coat, and finally the peritoneal covering are soon worn by the ulcerative process, causing perforation of the stomach and death by introducing into the peritoneum divers gases and alimentary matters, unless the effect of the perforation be anticipated and prevented by some providential adherence to surrounding viscera. The frequent perforation of the stomach as a consequence of this ulcer has suggested to professor Rokitanski the name by which he has designated it, viz: the perforating ulcer of the stomach.

The series of facts has enabled me to observe all the degrees of the perforating ulcer. In the first degree the ulceration is limited to the mucous membrane, and generally consists of an erosion of the follicles. In the second degree the fibrous tunic which is the true frame work of the stomach has been invaded and destroyed; we find at the bottom of the ulcer the muscular fibres which are exposed. In the third degree the muscular fibre has disappeared, and, of all the coats of the stomach, the peritoneal covering stands alone. In the fourth degree, the thickness of the stomach is destroyed, so that it may be replaced in that point by the surrounding organs with which it has contracted intimate adhesions.

The simple chronic ulcer of the stomach presents but a coarse resemblance to the cancerous ulcer, with which it is generally confounded. The indurated base around the former offers none of the attributes of the scirrhous or encephaloid cancer; we do not find that hypertrophy of surrounding tissues which almost always accompanies cancer, and which has been so often taken for the cancerous degeneration itself. Moreover, the best proof that the simple chronic ulcer of the stomach is not cancerous, is its curability. This curability of the simple ulcer of the stomach has also been proved by pathological anatomy, for it has shown the texture of the cicatrices resulting from the healing process, whereas those very cicatrices have been considered as belonging to the scirrhous cancer.

The first case of simple ulcer of the stomach which came under my observation had been sent to me as a case of cancer. The patient, who was a learned Greek scholar, had been sucessively treated by several physicians, who all supposed him to be affected with cancer of the stomach, as even, at the autopsy, the cicatrized ulcer was looked upon as scirrhous cancer.

It is, therefore, important to study the nature of the cicatrix resulting from the simple ulcer of the stomach. These cicatrices are made up of fibrous tissue, being constituted by a coating, more or less thick, of new fibres, covering the loss of substance. It is incorrect to say that the loss of substance in mucous membrane is replaced by another mucous membrane, of new formation. Those cicatrices have never offered the least structural characteristic of mucous membranes, no villi, no follicles are to be seen, unless some of these have been left standing by the ulceration. I have not had an opportunity of ascertaining if the cicatrix was covered by an epithelium of the same kind as that of the stomach. The mucous membrane ceases abruptly at the circumference in the form of a dense circular ridge.

The ulcers of the stomach heal in the same way as any loss of substance on the external tegument, viz: by a double mechanism. 1st, bringing together the edges of the ulcer by puckering them up, and thus diminishing the circumference of the abraded surface, which accounts for the radiated appearance of the cicatrix. And, secondly, the formation of an entirely new fibrous tissue constituting the cicatrix.

When the loss of substance is inconsiderable, it is repaired according to the first mechanism, and then the cicatrix in the stomach is represented by a line or a small loop with puckered edges and radiating folds. On the contrary, large abrasions leave a circular depression, as if made with a punch, with a fibrous centre, limited by a ridge, more or less elevated, formed by the mucous membrane, and quite distinct from the tissue of the cicatrix, the line of demarcation being well marked. The simple ulcer is sometimes seen, after having destroyed successively all the coats of the stomach, to pass the limits of that organ, and extend its ulceration to those adjoining tissues which have become adherent to the peritoneal covering of the stomach. Thus the pancreas is generally called upon to repair the breach in the stomach, because the posterior wall of the stomach is mostly the seat of the simple ulcer. Thus, also, the liver repairs the loss of substance in the anterior part of the lesser curvature. I have also seen the spleen stop up a hole which perforated the lesser extremity of the stomach, as also the transverse colon, replacing the corresponding part of the greater curvature.

In a case presented to the Anatomical Society by M. Barth, in the month of February, 1851, a perforation was seen in the anterior wall of the stomach measuring nearly an inch in diameter, but the loss of substance was replaced by the anterior wall of the abdomen, and by the posterior part of the sternum which was already attacked by the ulcer. The xiphoid appendage was thus posteriorly deprived of its periosteum, and even destroyed in some points, so that if this old woman, the subject of the lesion, had lived a little longer, the whole appendix would have been completely destroyed; the soft parts perforated by the ulcerative process, and thus a gastro-cutaneous fistula would have been produced. In another case which we observed, the missing parts had been replaced —first, above and in front of the liver, of which the color appeared through the cicatrix; second, behind by the pancreas, easily known by its granular appearance; third, in front and below by the arch of the colon, which was puckered at this place.

And not only do the viscera which are in the neighborhood of the stomach thus repair the loss of substance occasioned by ulceration, but having become a constituent part of the organ, they also participate in the ulcerative process, become eroded, and lose their own substance, and are finally perforated, if they are tubular organs. Thus it is that I have seen a simple chronic ulcer of the stomach, occupying the great curve of the stomach, open into the transverse colon; another ulcer of the same kind opened into the duodenum; and lastly, a simple ulcer of the stomach communicated with the diaphragn into the left bronchial tube.

*On the Subsequent Ulceration of the Cicatrix, produced by the simple Ulcer.*—A most important view under which the cicatrix of the stomach must be viewed is the facility with which they become the seat of a subsequent ulcerative process. The fibrous cicatricious tissue of the stomach, so different from the mucous membrane, by its want of vitality and its powers of resistance to the numerous causes of irritation to which it is exposed, is prone to inflammation, and once inflamed, ulceration soon follows, with all the symptoms of the simple ulcer; hence those frequent returns of the same disease, which I have seen take place one year, two years, five years, and even eight years after a cure that had appeared final. And thus, if the most severe treatment does not put a stop to the ulcerative process, the patient may die from perforation or hemorrhage. Perforation and hemorrhage are the principal accidents, and the great cause of danger arising from a simple ulcer of the stomach, and the danger continues even after the circatrix is formed, so that perforation or hemorrhage may take place either primitively, that is to say, during the period of ulceration, or consecutively, that is to say, after the formation of the cicatrix.

*Of the Spontaneous Perforation of the Stomach by simple Ulceration.*—The simple ulcer appears to me the most frequent cause of the spontaneous perforation of the stomach. On examining critically the principal observations which have been published on the spontaneous perforation of that organ, I have easily recognized in the description of the post-mortem appearances all the characters of the simple acute, or chronic ulcer of the stomach. Such, for instance, is the case recorded of that learned chemist d'Arcet, who at 72 years of age, after suffering during six months from difficult digestion, died from a spontaneous perforation of the stomach which occurred after a light meal.

These rapidly fatal accidents which are the consequence of perforation of the stomach come on very suddenly and sometimes immediately after taking into the stomach some food or drink, so that it has often given rise to a suspicion of poison.

The most remarkable case of the kind which I have ever seen, is that of a coal merchant, a man of athletic frame, age 23 years, who, while carrying a bag of coal, stopped on his way at a wine merchant's to drink a glass of wine; after a few minutes, he was taken with the most violent colic; he received the first medical assistance at his own house, but was carried the next day in a dying state to the Maison de Sante, faubourg, St. Denis, where I was at the time assistant to our venerable colleague Mr. Dumeril. This was on the 5th of December, 1829. The patient presented all the symptoms of peritonitis, resulting from perforation and died in full possession of his reason three hours after entering the hospital. I had, however, obtained from himself an important piece of information, viz: that he had been suffering from his stomach for some months, and that his digestion was very tedious. The fraternity of coal merchants were convinced that their comrade had been poisoned, and that the glass of wine he had taken immediately before the accident was the vehicle which contained the poison; they immediately determined to prosecute the wine merchant, and desired that the post-mortem examination should be made before a deputation of their order.

It turned out to be, as I had previously announced, a case of spontaneous perforation by simple ulceration of the stomach. A remarkable circumstance was that this ulcer, occupying the pyloric extremity of the stomach, was in the form of a zone. The centre of the ulceration had invaded the muscular fibres of the stomach, and the perforation had taken place at a point where those fibres were destroyed in such a manner that the coats of the stomach reduced to their peritoneal covering had given way under a slight effort.

This is an example of primitive perforation by the simple ulcer of the stomach, by which I mean that the perforation occurred during the progressive stage of the ulcer; but consecutive perforation may also happen when the ulcer is completely cicatrized. I may even say that the latter accident is the most frequent, so that we sometimes observe a chronic ulcer completely cicatrized except in a single point where the ulceration continued, or sometimes the ulcerative process invades an old cicatrix which is destroyed with more or less rapidity.

We must not confound the latter case with that of a new ulcer which may be located near an old scar.

I consider as true the following proposition, viz: the spontaneous preforation of the stomach is incomparably more frequent in simple ulcerations of the stomach than in the cancerous form; in fact, perforation in cancer of the stomach is a most rare occurrence if it has ever been observed.

I now pass to the second class of accidents from simple ulcer of the stomach, viz:

*Hemorrhage.*—We may divide hemorrhages resulting from the simple ulceration of the stomach into two classes; that is to say, it is *primitive* when it occurs during the period of progress of the ulcer, it is *consecutive* when it takes place after the complete formation of the cicatrix. We might also divide hemorrhages of the stomach according to the quantity of blood lost, which may be *small*, *moderate* or fatal.

A small hemorrhage is almost inevitable in the simple ulcer of the stomach, either in its acute or chronic stage, until cicatrization takes place. If we examine under the surface of pure water, either with the naked eye or with a magnifying glass, the appearance of a simple ulcer of the stomach, we shall see a number of small vessels open, with their edges smoothly cut, some of them being stopped by solid clots firmly adherent, others on the contrary containing soft clots which are evacuated by the slightest touch. The latter give rise to those small but repeated hemorrhages in consequence of which the blood thus poured out is mixed with the food, and produces those dark colored stools, or the black vomitings which are often the first revealing symptoms of the disease. But it sometimes happens that the simple ulcer of the stomach comes in contact with a large artery, destroys its coats and perforates the vessel; then unless a very solid clot prevents the blood from being poured out, the vomitings and the passages may contain pure blood, in various quantities according to the size of the vessel, and thus hemorrhage may become rapidly fatal.

I have seen a case in which the patient died from hemorrhage without discharging a single drop of blood, but at the post-mortem examination I found the stomach distended by an enormous quantity of coagulated blood, the large and small intestines being equally full. A perforation of the splenic artery had been the cause of hemorrhage. Patients affected with this disease generally die from the repeated vomitings, or the frequent bloody passages, which reduce them to the last degree of anemia and marasmus.

And here I must repeat, what I have already said relative to perforation, viz: that death by hemorrhage is much more frequent as a consequence of the simple ulcer than of the cancerous affection of the stomach.

The yellow elastic tissue or proper tunic of the arteries escapes many organic diseases by its very want of vitality, resisting at times even cancer itself, but it cannot withstand the disorganizing process so aptly called (by Hunter,) *ulcerative inflammation*, for that respects no tissue, and is nothing more than a species of erosion, or molecular gangrene, similar to that produced by the corrosive action of different liquids, destroying layer after layer in concentric zones. We not unfrequently see an ulcer of the stomach perfectly healed, except in a point corresponding to some large arterial vessel. Now, according to the law that all loss of substance in an artery can be permanently cured only by an entire obliteration, it may happen that if the clot is not firmly adherent it may detach itself and bring on hemorrhage sufficient to cause immediate death.

Sometimes again the ulcerative process stops at the external surface of an artery of which the inner tunics are yet intact; then cicatrization having taken place, the arterial vessel is in a manner included in the cicatrix.

Now if the ulcerative inflammation happens to seize upon this cicatrix, one of its first effects will be the erosion and perforation of the artery, another way again of producing fatal hemorrhage.

The ordinary source of those frightful hemorrhages from the stomach consequent upon simple ulceration, is the splenic artery, a large vessel which is situated behind the stomach, along the superior border of the pancreas where it describes a serpentine course, which brings it in contact with the stomach in many points.

I have also seen a fatal case of gastrorrhagia produced by the perforation of the coronary artery of the stomach. The size of the gastroepiploic arteries is sufficient to cause death in case they be perforated, but we know that the simple ulcer seldom occupies the greater curvature of the stomach.

In order to complete the history of this disease, we have yet to examine its clinical characters, and means of diagnosis, and also the therapeutical agents by which it can be treated successfully. Those points will form the subject of another lecture.—*New Orleans Med. News and Hospital Gazette.*

